# Peptide functionalized liposomes for receptor targeted cancer therapy

**DOI:** 10.1063/5.0029860

**Published:** 2021-01-25

**Authors:** Matthew R. Aronson, Scott H. Medina, Michael J. Mitchell

**Affiliations:** 1 Department of Bioengineering, University of Pennsylvania, Philadelphia, Pennsylvania 19104, USA; 2 Department of Surgery, Division of Otolaryngology, Children's Hospital of Philadelphia, Philadelphia, Pennsylvania 19104, USA; 3 Department of Biomedical Engineering, The Pennsylvania State University, University Park, Pennsylvania 16802, USA; 4 Huck Institutes of the Life Sciences, The Pennsylvania State University, University Park, Pennsylvania 16802, USA; 5 Abramson Cancer Center, Perelman School of Medicine, University of Pennsylvania, Philadelphia, Pennsylvania 19104, USA; 6 Institute for Immunology, Perelman School of Medicine, University of Pennsylvania, Philadelphia, Pennsylvania 19104, USA; 7 Cardiovascular Institute, Perelman School of Medicine, University of Pennsylvania, Philadelphia, Pennsylvania 19104, USA; 8 Institute for Regenerative Medicine, Perelman School of Medicine, University of Pennsylvania, Philadelphia, Pennsylvania 19104, USA

## Abstract

Most clinically approved cancer therapies are potent and toxic small molecules that are limited by severe off-target toxicities and poor tumor-specific localization. Over the past few decades, attempts have been made to load chemotherapies into liposomes, which act to deliver the therapeutic agent directly to the tumor. Although liposomal encapsulation has been shown to decrease toxicity in human patients, reliance on passive targeting via the enhanced permeability and retention (EPR) effect has left some of these issues unresolved. Recently, investigations into modifying the surface of liposomes via covalent and/or electrostatic functionalization have offered mechanisms for tumor homing and subsequently controlled chemotherapeutic delivery. A wide variety of biomolecules can be utilized to functionalize liposomes such as proteins, carbohydrates, and nucleic acids, which enable multiple directions for cancer cell localization. Importantly, when nanoparticles are modified with such molecules, care must be taken as not to inactivate or denature the ligand. Peptides, which are small proteins with <30 amino acids, have demonstrated the exceptional ability to act as ligands for transmembrane protein receptors overexpressed in many tumor phenotypes. Exploring this strategy offers a method in tumor targeting for cancers such as glioblastoma multiforme, pancreatic, lung, and breast based on the manifold of receptors overexpressed on various tumor cell populations. In this review, we offer a comprehensive summary of peptide-functionalized liposomes for receptor-targeted cancer therapy.

## I. INTRODUCTION

Liposomes have been at the forefront of drug delivery research for the past few decades, following their discovery in 1965 by Alec Bangham.^1,2^ Since then, they have been delineated into categories including small unilamellar vesicles (SUVs < 100 nm), medium unilamellar vesicles (MUVs 100–250 nm), large unilamellar vesicles (LUVs > 250 nm), and giant unilamellar vesicles (GUVs)—with the majority of drug delivery studies focusing on SUVs.^3^ Composed of a concentric hydrophobic phospholipid bilayer compartmentalizing an aqueous core from its aqueous environment, liposomes offer a plethora of controlled delivery applications for different classes of drugs.^4^ In brief, hydrophobic drugs such as small molecule chemotherapeutics can be loaded into the lipid lamella, while hydrophilic therapies such as nucleic acids can be loaded into the aqueous core for applications such as gene editing.^5,6^ The majority of current cancer therapies rely on the systemic administration of chemotherapeutic agents that exhibit off-target effects due to their inability to differentiate between healthy and tumor tissue—this often results in side effects like nausea and fatigue or more severely cardiotoxicity.^7–10^ To improve the therapeutic index of these agents, drugs can be encapsulated in liposomal membranes, which act as a barrier to decrease toxic effects in healthy tissues. Several liposomal drug formulations have been approved by the FDA, loading chemotherapeutics such as doxorubicin (Doxil), duanorubicin (DaunoXome), cytarabine (Depocyte), vincristine (Marqibo), mifamurtide (Mepact), irinotecan (Onivyde), and daunorubicin/cytarabine (Vyxeos) (Table I).^11–15^ Some of these nanoparticle drug formulations also take advantage of PEGylation, which is thought to act as a nanoparticle cloak to greatly enhance their circulation time and reduce immune responses.^16,17^

**TABLE I.** Clinically approved liposomal cancer therapies.

Name	Encapsulated drug	Indications	Year approved	Ref.
Doxil/Caelyx	Doxorubicin	HIV-related Kaposi's sarcoma	1995	11, 12, 14, 25
		Ovarian cancer	2005	
		Multiple myeloma	2008	
		Breast cancer	2012	
DaunoXome	Duanorubicin	HIV-related Kaposi's sarcoma	1996	
Depocyt	Cytarabine/Ara-C	Neoplastic meningitis	1999	
Myocet	Doxorubicin	Metastatic breast cancer	2000	
Mepact	Mifamurtide	High-grade, resectable, and non-metastatic osteosarcoma	2004	
Marqibo	Vincristine	Acute lymphoblastic leukemia	2012	
Onivyde	Irinotecan	Metastatic pancreatic cancer	2016	
Vyxeos	Daunorubicin and Cytarabine	High-risk acute myeloid leukemia	2017	13

Relying on passive localization of nanoparticles to target tissue has many limitations. When in the bloodstream, liposomes are immediately coated with a surface of plasma proteins that form a protein corona.^18^ Circulation times can often be modulated by the protein species present in the corona, leading to either extended circulation times due to dysopsonins, or more often limiting it through rapid clearance by the reticuloendothelial system (RES) or mononuclear phagocyte system (MPS) with opsonins.^19,20^ PEGylation of liposomes was thought to circumvent these limitations by shielding protein coronas from forming and subsequently undergoing clearance, but recent studies have shown contradicting evidence with a phenomenon called accelerated blood clearance (ABC) and complement activation-related pseudoallergy (CARPA).^21–24^ Furthermore, tumor localization heavily relies on the enhanced permeability and retention (EPR) effect, which is the combined phenomenon of leaky vasculature proximal to the tumor and blocked lymphatic drainage within the tumor microenvironment. Unfortunately, although this phenomenon is repeatedly observed in mice, it is highly variable in human patients in part due to cancer heterogeneity.^26–30^ Therefore, active targeting of tumor tissue through functionalization of liposomes offers an avenue to greatly enhance localization of therapies to minimize collateral damage.^31^

**FIG. 1.**
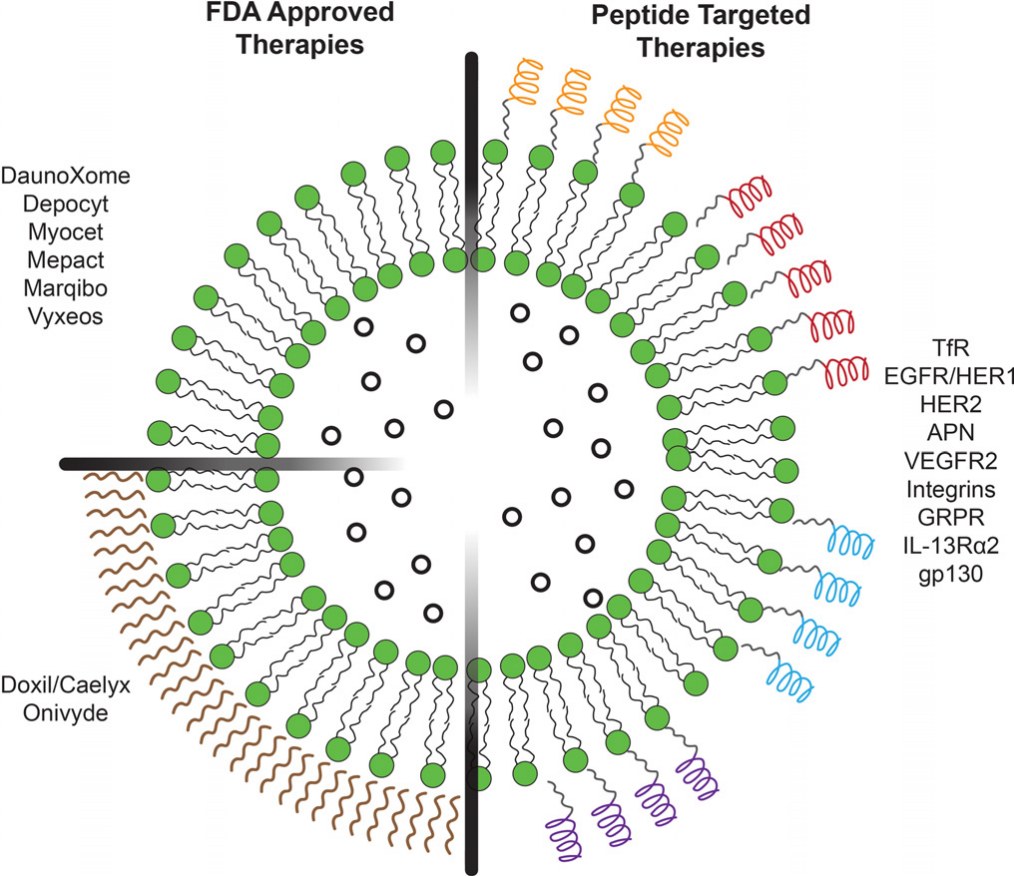
Schematic illustration of liposome-encapsulated therapies. (Left) FDA-approved therapies for chemotherapeutic-encapsulated liposomes (top left) without PEGylation and (bottom left) with PEGylation. (Right) Peptide-functionalized liposomes for receptor-targeted chemotherapeutic delivery.

There are several options for modifying liposomes to enable tumor targeting,^30^ as many groups have explored the use of peptides, proteins and antibodies,^31–38^ nucleic acids and aptamers,^33–36^ and carbohydrates.^33,39–42^ Each of these biomolecules offers advantages and disadvantages in applicability, such as high biocompatibility and bioactivity, as either liposome-membrane fusogens^43,44^ or ligands for receptor targeting.^25,30^ Here, we discuss peptides because they are ideal for liposomal surface modification due to their ease of synthesis, ease of manufacturing at industrial and clinical levels, and chemical versatility.^47–49^ Overall, these advantageous characteristics promote an immense field for novel innovation and discovery for a plethora of diseases. In this review, we focus on the recent advancements in receptor targeting peptide-functionalized liposomes over the past five years. By peptides acting as ligands with high affinities toward overexpressed cell membrane receptors, liposomes encapsulating chemotherapeutic agents can improve targeting *in vitro* and *in vivo* and act as clinically applicable therapies for improved cancer treatment (Fig. 1).

## II. RECEPTOR-TARGETING PEPTIDE LIGANDS

One avenue to actively target cancer cells is through receptor–ligand binding. Many novel peptides have been discovered to act as ligands with high affinity for a plethora of overexpressed receptors on the surface of cancer cells. Therefore, functionalizing liposomes with these sequences offers a method of tumor targeting, with a simultaneous reduction in off-target toxicity. The receptors focused on in this section include the transferrin (TfR), epidermal growth factor (EGFR/HER1 and HER2), gastrin-releasing peptide (GRPR), aminopeptidase N (APN), vascular endothelial growth factor 2 (VEGFR2), and integrin receptors (Table II).

**TABLE II.** Peptide ligands for targeting tumor and tumor microenvironment receptors. ^L^ denotes L-amino acids, and ^D^ denotes D-amino acids.

Targeted receptor	Peptide name	Peptide sequence	Reported K_D_ (M)	Ref
TfR	T7	HAIYPRH	2.1 × 10^−8^	50, 51
EGFR/HER1	GE11	YHWYGYTPQNVI	2.2 × 10^−8^	52–54
HER2	P6.1	KCCYSL	3.0–4.5 × 10^−8^	55
	HER-2 Peptide	YCDGFYACYMDV	—	56
	AHNP	FCDGFYACYADV	3.59 × 10^−7^	57
APN	NGR	NGR	—	58
	LN	YEVGHRC	1.0 × 10^−7^	59
VEGFR2	STP	SKDEEWHKNNFPLSP	8.50 × 10^−8^	60, 61
	TP	TIDHEWKKTSFPLSF	5.93 × 10^−7^	60
	S1	LIDHEWKENYFPLSF	1.31 × 10^−7^	62
	^L^A7R	ATWLPPR	9.29–18.09 × 10^−9^	63–66
	^D^A7R	ATWLPPR	8.41 × 10^−9^	63
	Cyclic A7R	CATWLPPR	6.79 × 10^−9^	66
Integrins	Linear RGD	RGD	—	67–72
		GRGDS	—	73
	Cyclic RGD	c(RGDyC)	—	74
		c(RGDfK)	—	75
		c(RGDf[*N*-methyl]C)	—	76
		c(RGDyK)	—	77, 78
	RWrNK	RWrNK	1.6 × 10^−9^	79
	P1c	CIRTPKISKPIKFELSG	—	80, 81
GRPR	Cystabn	FQWAVGH-Sta-L-NH_2_	—	82
IL-13Rα2	Pep-1	CGEMGWVRC	—	83
gp130	VTW	VTWTPQAWFQWV	—	84

When functionalizing the surface of liposomes with peptides, there are two general strategies for conducting this modification. First, ligands can be covalently conjugated to lipid headgroups or polymer extensions (such as PEG) that project the ligand orthogonally from the liposomal surface.^19^ More specifically, it is generally understood that there exist four main chemical conjugation methods to achieve these modifications: activated carboxyl groups can react with amino groups to form amide bonds, pyridyldithiols can react with thiols to form disulfide bonds, maleimide derivatives can react with thiols to yield thioether bonds, and p-nitrophenylcarbonyl groups can react with amino groups to form carbamate bonds.^30^ Alternatively, peptides can adsorb to and/or interpolate into the liposomal surface via electrostatic and/or hydrophobic interactions.^19,45^ Both these methods present the ligands to their respective receptors and aid in the targeting of specific tumor cells.

### A. Transferrin receptor (TfR)

**FIG. 2.**
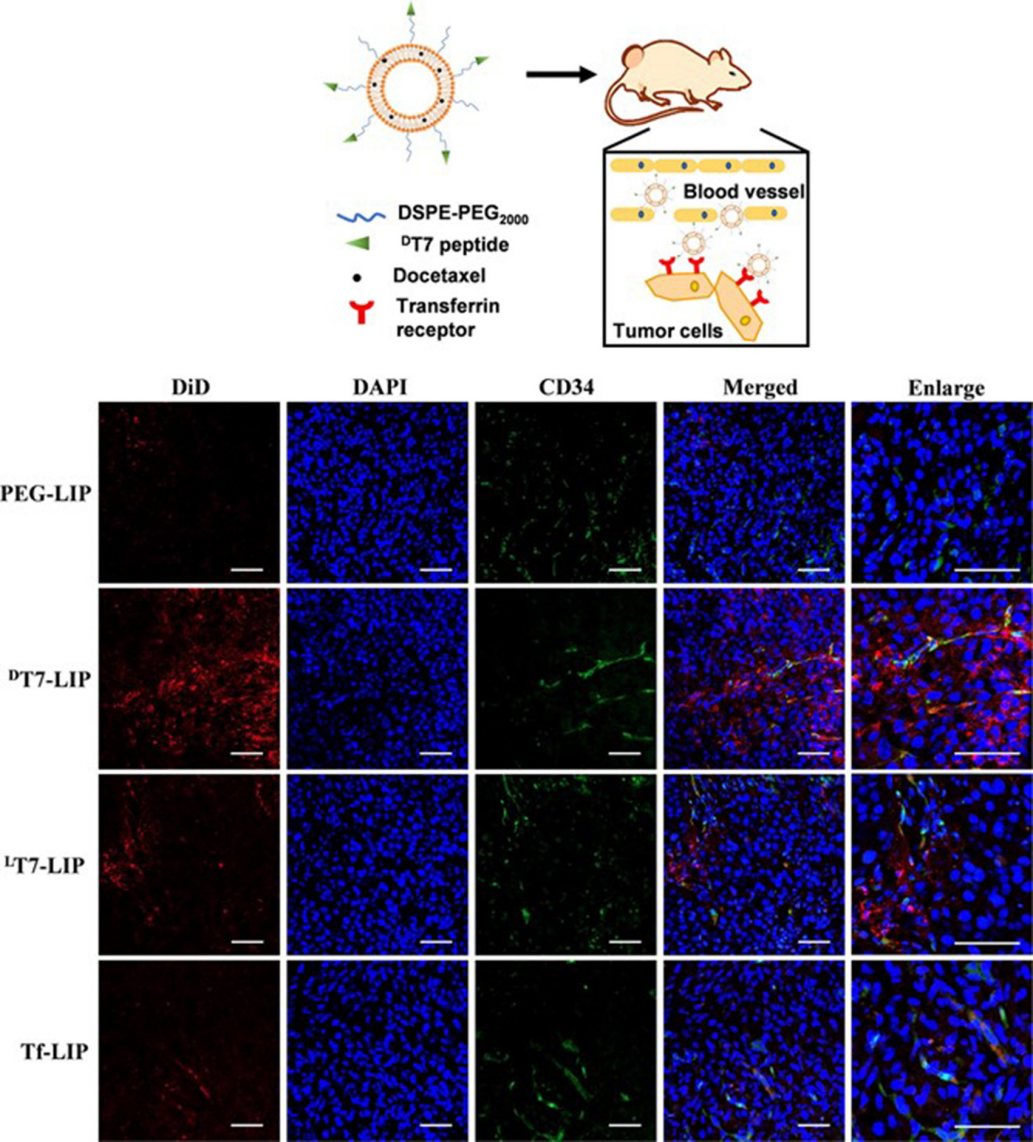
Intratumoral distribution of liposomes encapsulated with DiD in HepG2 xenograft tumors. (Red) DiD-encapsulated liposome. (Blue) DAPI-cell nuclei. (Green) anti-CD34-blood vessels. Scale bar = 40 *μ*m. Reprinted with permission from Tang *et al.*, “A stabilized retro-inverso peptide ligand of transferrin receptor for enhanced liposome-based hepatocellular carcinoma-targeted drug delivery,” Acta Biomater. **83**, 379–389 (2019). Copyright 2019 Elsevier.

Transferrin receptors (TfRs) are often overexpressed in a variety of cancer types due to the increased metabolic demand for iron, indicating an attractive targeting receptor for cancer therapeutics.^85,86^ Peptide T7, sequence HAIYPRH, has been widely characterized and shown to exhibit a high binding affinity to TfR. One group compared peptide-modified liposomes to target hepatocellular carcinoma cells with L- and D-enantiomers, ^D^T7 and ^L^T7, as well as a transferrin (Tf)-modified liposome control.^50^ It would be expected for the D-enantiomer to exhibit a decreased selectivity due to chiral specific receptors; however, they demonstrated increased binding affinity via surface plasmon resonance (SPR) and *in vitro* studies where ^D^T7-modified liposomes greatly enhanced the targeting ability of liposomes to the cells over ^L^T7 and Tf. Furthermore, the results were replicated *in vivo* with a significant reduction in tumor growth in mice treated with ^D^T7-modified liposomes loaded with the chemotherapeutic docetaxel. Upon tumor sectioning of 24 h post-injection mice with DiD dye-loaded liposomes, immunofluorescence shows a significant increase in tumor accumulation when nanoparticles are decorated with ^D^T7 (Fig. 2). Another group focused on treating lung cancer and used T7-functionalized liposomes loaded with quercetin to improve localization.^43^ Their results show that the T7 surface-modified liposomes increased the induction of apoptosis significantly compared to all controls and improved localization of drug-loaded nanoparticles toward A549 cells compared to MRC-5 normal lung fibroblasts. Remarkably, they demonstrated a threefold increase in cytotoxicity of quercetin when loaded into T7-functionalized liposomes compared to the free drug. Importantly, tumor penetration was visualized with fluorescently labled liposomes and quantified to find a significant increase in the penetration depth with T7 surface modifications. Altogether, ^D^T7 offers not only advantages over ^L^T7 due to proteolytic stability but also enhanced tumor targeting, advancing the field of TfR-specific cancer therapeutics.

### B. Epidermal growth factor receptor (EGFR)

Erythroblastic leukemia viral oncogene homolog (ERBB) receptors are a family of four transmembrane receptor tyrosine kinases (RTKs) that are often overexpressed during the malignant transformation of healthy cells.^87,88^ The four receptors within this family include epidermal growth factor receptor (EGFR) or HER1, as well as HER2, HER3, and HER4. In 2005, the peptide sequence YHWYGYTPQNVI, known as GE11, was designed using phage display by the labs of Yuhong Xu and Jianren Gu and exhibited targeting affinities for EGFR.^52,53^ Recently, this sequence became used for liposomal conjugation to exploit its EGFR-targeting capabilities. In 2008, Song *et al.* showed the preliminary targeting capabilities of GE11-functionalized, unloaded liposomes.^89^ In 2014, Cheng and colleagues functionalized liposomes encapsulating doxorubicin (Dox) and demonstrated increased drug delivery with a 2.6-fold reduction in IC_50_ of the modified particles compared to the nontargeted control.^54^ Further, tumor accumulation was exhibited by a 2.2-fold greater fluorescence intensity of the GE11 liposomes compared to the untargeted control. More recently, in 2017, Xu *et al.* focused on the localized delivery of docetaxel (DTX) and an siRNA responsible for silencing the ABCG2 gene regulating multidrug resistance (MDR).^52^ *In vitro*, they showed a reduction in the IC_50_ of DTX in Hep-2 laryngeal cancer cells when treated with GE11 liposomes compared to treatment with free drug. Furthermore, *in vivo* Hep-2 xenograft-bearing mice demonstrated that the GE11 liposomes greatly enhanced tumor growth inhibition compared to the non-targeted liposomes.

Ringhieri and coworkers designed liposomes coated with the HER2-targeting peptide P6.1, sequence KCCYSL, for the treatment of breast cancer.^55^ Liposomes were modified with the monomers, dimers, and tetramers and treated against the under- and over-expressing HER2 breast cancer cells MDA-MB-231 and BT-474, respectively. The results showed that tetrameric P6.1-conjugated liposomes had a 10 times greater degree of cellular binding and uptake in BT-474 compared to MDA-MB-231 cells, highlighting their targeting ability to cells with overexpressed HER2. Furthermore, the tetrameric peptide yielded greater binding and uptake than other peptide forms. Gratifyingly, the levels present were comparable to that of the clinically approved anti-HER2 antibody Herceptin. Often, peptides are compared against Herceptin as the current standard of localization to HER2.^55,56^ Using the antibody as a template, Shi *et al.* synthesized an HER-2 peptide analog, sequence YCDGFYACYMDV, which showed increased tumoral delivery of doxorubicin-loaded and pH-sensitive liposomes toward multidrug-resistant MCF-7 breast cancer cells.^56^ In this study, they showed a strong increase in mitochondrial localization compared to other organelles, which is important for increasing mitochondrial-driven apoptosis. Zahmatkeshan and coworkers also studied HER2 targeting with a different peptide and Anti-HER2/neu peptide (AHNP) with sequence FCDGFYACYADV, this time to improve localization of doxorubicin-loaded PEGylated liposomes.^57^ Their results demonstrated that increasing the density of functionalized peptides significantly increases tumor homing and uptake in two HER2 overexpressing lines, SKBR3 and TUBO. When tested in a TUBO breast cancer mouse model, liposomes functionalized with 100 and 200 ligands showed a similar tumor growth delay and a significantly longer life expectancy.

### C. Aminopeptidase N (APN)

Aminopeptidase N (APN) has been studied extensively due to its overexpression on the surface of cancer cells, most commonly seen in aggressively growing phenotypes. APN is a zinc metalloenzyme found in the plasma membrane, responsible for cleaving N-terminal neutral amino acids; this can lead to functions relating to migration and invasion or metastasis in tumor cells.^90^ Therefore, exploitation of this overexpression can lead to tumor targeting of nanoparticles. A widely studied peptide known to selectively bind to APN is the tripeptide sequence NGR, which has been appended onto the surfaces of liposomes to target multiple tumor types.^91–93^ Huang and collaborators presented an NGR-modified liposome for improved glioma targeting and the delivery of combretastatin A4.^58^ Their formulation showed success in targeting *in vitro* when treating U87-MG human glioma tumor cells for the inhibition of cellular migration and reduction in vasculogenic mimicry (VM). Subsequently, *in vivo* results with U87-MG orthotopic tumor-bearing mice demonstrated enhanced targeting of cancer cells with both anti-tumor and anti-VM activities.

Jia *et al.* utilized a novel peptide with a high affinity for APN called LN, sequence YEVGHRC, to functionalize liposomes loaded with doxorubicin to treat HepG2 cells.^59^ *In vitro*, LN conjugation greatly enhanced the cell internalization of doxorubicin-loaded liposomes; these results were reproduced *in vivo* in a subcutaneous HepG2 xenograft BALB/c nude mouse model with significantly greater tumor accumulation and decreased tumor growth. The implications of peptide-modified liposomes to target APN, particularly with novel sequences such as LN, are increasing in importance paralleling the rise in aggressive and drug-resistant tumors.

### D. Vascular endothelial growth factor receptor 2 (VEGFR2)

**FIG. 3.**
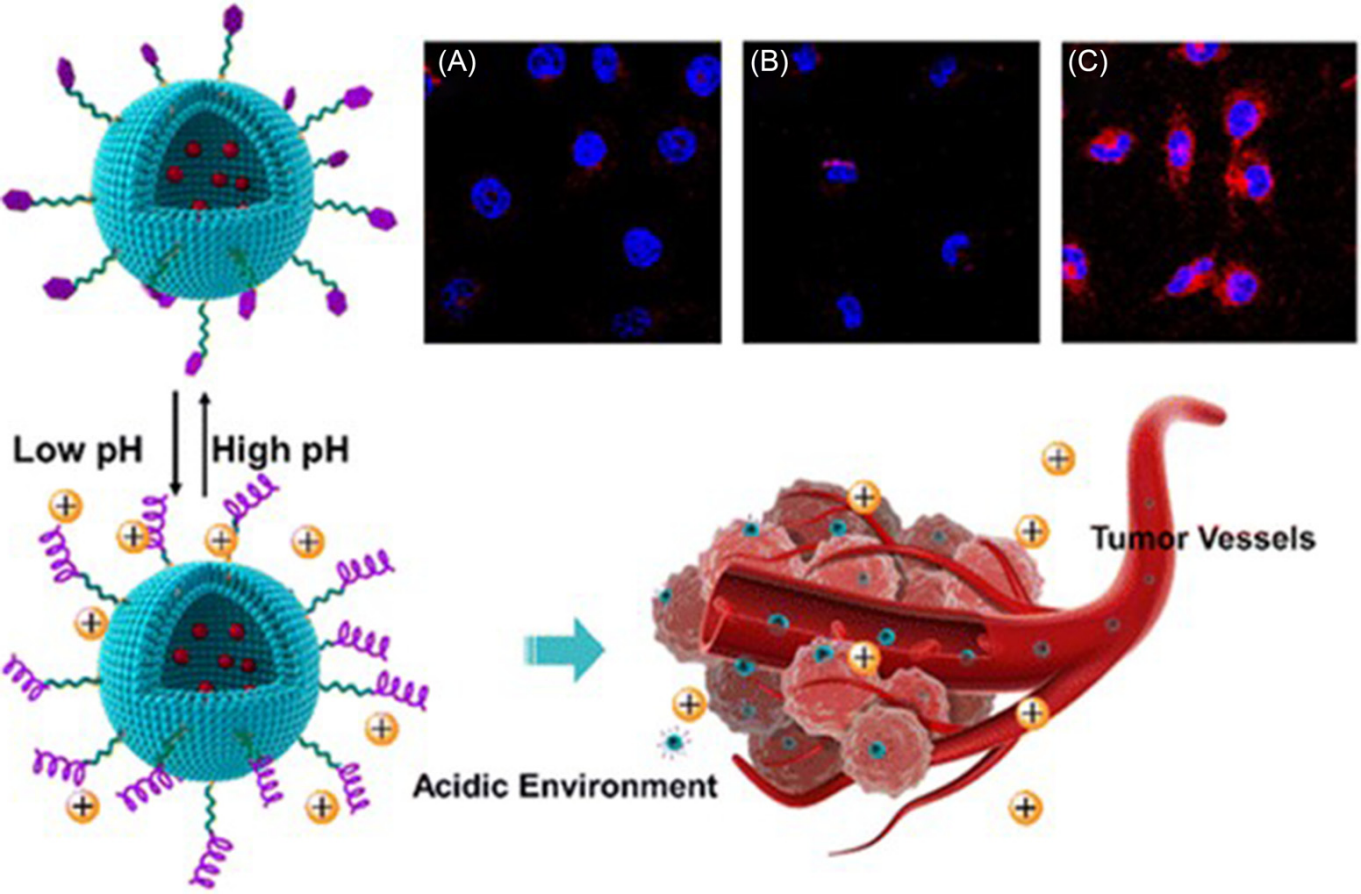
Schematic illustration of STP-decorated, doxorubicin-loaded liposomes in response to pH changes for targeting the tumor microenvironment. Confocal micrographs of HUVEC cells with stained nuclei (blue) and doxorubicin (red) treated with (a) STP-LS-DOX at pH 7.4, (b) LS-DOX at pH 5.8, and (c) STP-LS-DOX at pH 5.8. Adapted with permission from Han *et al.*, ACS Appl. Mater. Interfaces **8**(29), 18658–18663 (2016). Copyright 2016 American Chemical Society.

Vascular endothelial growth factor receptor 2 (VEGFR2) is a well-characterized and overexpressed angiogenesis marker commonly found in newly formed tumor vessels.^94–96^ Activation of this tyrosine kinase receptor has been shown to increase proliferation and migration and subsequently lead to the metastasis of tumors. Zhigyuan Hu's group has investigated peptides that can target VEGFR2 for functionalizing liposomal nanoparticles and directing them to the tumor microenvironment.^60–62^ Qian *et al.* first used an imprinting microarray to optimize a peptide sequence to accomplish this, arriving at the peptide STP with sequence SKDEEWHKNNFPLSP.^60^ STP became activated in low pH tumor microenvironments by conforming to an alpha-helix with the tri-amino acid section Asp–Glu–Glu enabling strong binding to VEGFR2. *In vivo*, STP successfully recognized and penetrated human umbilical vein endothelial cells (HUVECs) in the tumor microenvironment. Another peptide characterized by Qian was TP, with the sequence TIDHEWKKTSFPLSF, which exhibited similar VEGFR2 targeting at a neutral pH compared to non-VEGFR2 overexpressing cells. Han *et al.* utilized the more successful STP peptide and used it to functionalize liposomes loaded with doxorubicin (STP-LS-DOX) for pH-responsive targeted drug delivery (Fig. 3).^61^ STP-LS-DOX exhibited limited HUVEC internalization at a neutral pH [Fig. 3(a)] and without the targeting peptide (LS-DOX) [Fig. 3(b)], but demonstrated significant accumulation in acidic conditions [Fig. 3(c)]. Furthermore, STP-LS-DOX was able to target HT-29 colon adenocarcinoma xenografts in mice through VEGFR2 targeting in tumor endothelial cells, and achieved high targeting efficiency indicated by significant induction of apoptosis of the tumor compared to controls. Further refinement of STP via microarray screening led to a second-generation peptide sequence with high affinity for VEGFR2 called S1, sequence LIDHEWKENYFPLSF.^62^ Using SPR imaging, S1 was determined to exhibit a K_D_ value of 131 nM, which is comparable to the VEGFR2 poly-antibody. Furthermore, S1 was able to preferentially localize to VEGFR2-overexpressing cells (HUVECs) over non-VEGFR2-expressing cells (293 T). When conjugated to doxorubicin-loaded liposomes, the inhibitory effects of doxorubicin were significantly higher in VEGFR2-overexpressing cells, demonstrating preferential localization. Moreover, the targeted distribution was reproduced in HT-29 tumor-bearing mice.

The peptide A7R, which was discovered by Binétruy-Tournaire and coworkers in 2000 via phage display, has been well characterized to bind with high affinity to VEGFR2 and neuropilin-1 (NRP-1).^97,98^ To improve stability and efficacy of the peptide, sequence ATWLPPR, in targeting tumors, modifications were made to A7R with great success. Cao *et al.* conjugated cysteine to the N-terminus of A7R to form A7RC and used it to modify paclitaxel-loaded liposomes; MDA-MB-231 breast cancer xenografts *in vivo* showed improved accumulation of the chemotherapeutic and greater inhibitory effects compared to the unmodified liposome control.^64^ Ying and colleagues further improved the efficacy and stability of the peptide through cyclization of the sequence (cA7R)^66^ and incorporation of D-amino acids,^63^ respectively. Cysteine was conjugated to A7R at the N-terminus to create an amide bond, and it was shown to bind with high affinity to VEGFR2 with a K_D_ value of 6.79 nM.^66^ When tested *in vitro* in VEGFR2-overexpressing endothelial (HUVEC) and glioma (U87) cells, fluorescein-labeled cA7R showed over 80% and 90% positive targeting and internalization, respectively. The localization results were recapitulated *in vivo* with U87 xenograft tumors, demonstrating improved localization of cA7R compared to linear A7R. Furthermore, cA7R-conjugated liposomes encapsulating doxorubicin showed significantly improved tumor volume reduction compared to nanoparticles with the linear peptide, doxorubicin, or doxorubicin alone. In parallel studies, ^D^A7R-modified liposomes were loaded with doxorubicin and used to treat subcutaneous tumor models. Using the D-enantiomer demonstrated increased proteolytic stability, significantly inhibited tumor growth, and significantly greater intracellular accumulation of the chemotherapeutic compared to the L-enantiomer or liposomal control.^63^ Most recently, the group modified myristic acid to ^D^A7R to improve blood–brain barrier (BBB) penetration in treating glioma; the results showed that when conjugated to liposomes, there was increased cell internalization, tumor and angiogenesis homing, and improved therapeutic outcome of the doxorubicin treatment.^65^

Important findings from these studies targeting VEGFR2 are the profound binding affinities seen with the sequences STP, S1, and A7R. When peptides have a K_D_ value within the same order of magnitude as antibodies, it is reasonable to expect selective binding activity that would significantly help localize modified liposomes to specific tumor cells for anticancer drug delivery. As seen with many of these peptides, there has been a surprising increase in targeting capabilities when switching the chirality to D-enantiomers; this area of research should be further explored to elucidate possible mechanisms for this activity.

### E. Integrin α_v_β_3_ and α_5_β_1_ receptors

Integrin α_v_β_3_ and α_5_β_1_ receptors are common targets on the cell membrane of cancer cells, due to their increased expression which contributes to cell adhesion, motility, invasion, and metastasis.^99,100^ The tripeptide RGD motif, arginine–glycine–aspartic acid, is perhaps the most common sequence to achieve high binding affinity and high selectivity. Many studies have been conducted, and reviews have been written on utilizing the linear RGD peptide and cyclic analogs.^25,101–103^ Below, we briefly describe the most recent advances made in both these categories, as well as sequences that target integrins not related to the RGD motif.

#### 1. Linear RGD

Linear RGD has been one of the most widely studied ligand appended to liposomes for improved tumor delivery. Zuo *et al.* functionalized docetaxel-loaded liposomes,^67^ Sonali *et al.* docetaxel and quantum dot-loaded liposomes,^68^ Wen *et al.* shikonin-loaded liposomes,^69^ and Tang *et al.* gemcitabine-loaded liposomes.^70^ Other groups focus on including pH-responsive materials to increase specificity in the acidic tumor microenvironment. Zhang and colleagues co-functionalized liposomes with RGD and a pH-responsive antimicrobial peptide ^D^H_6_L_9_ to selectively target tumor spheroids.^71^ Veneti *et al.* incorporated a pH-triggered elastin-like peptide linker (VPGVG)_n_ between the liposome and the RGD ligand to enhance peptide-cell interactions in acidic solutions.^72^ Parallel research effort has inserted spacers between the peptide ligand and the liposomal surface and studied its effect on peptide-receptor affinity. While most use a conventional PEG spacer, the potential immunogenicity of PEG polymers has urged the exploration of alternative linkers. For example, Veneti *et al.* used (VPGVG)_n_ that acted as a pH-responsive linker,^72^ while Suga *et al.* used the linker (SG)_n_ to greatly improve not only targeting but also intratumoral distribution.^73^

#### 2. Cyclic RGD

**FIG. 4.**
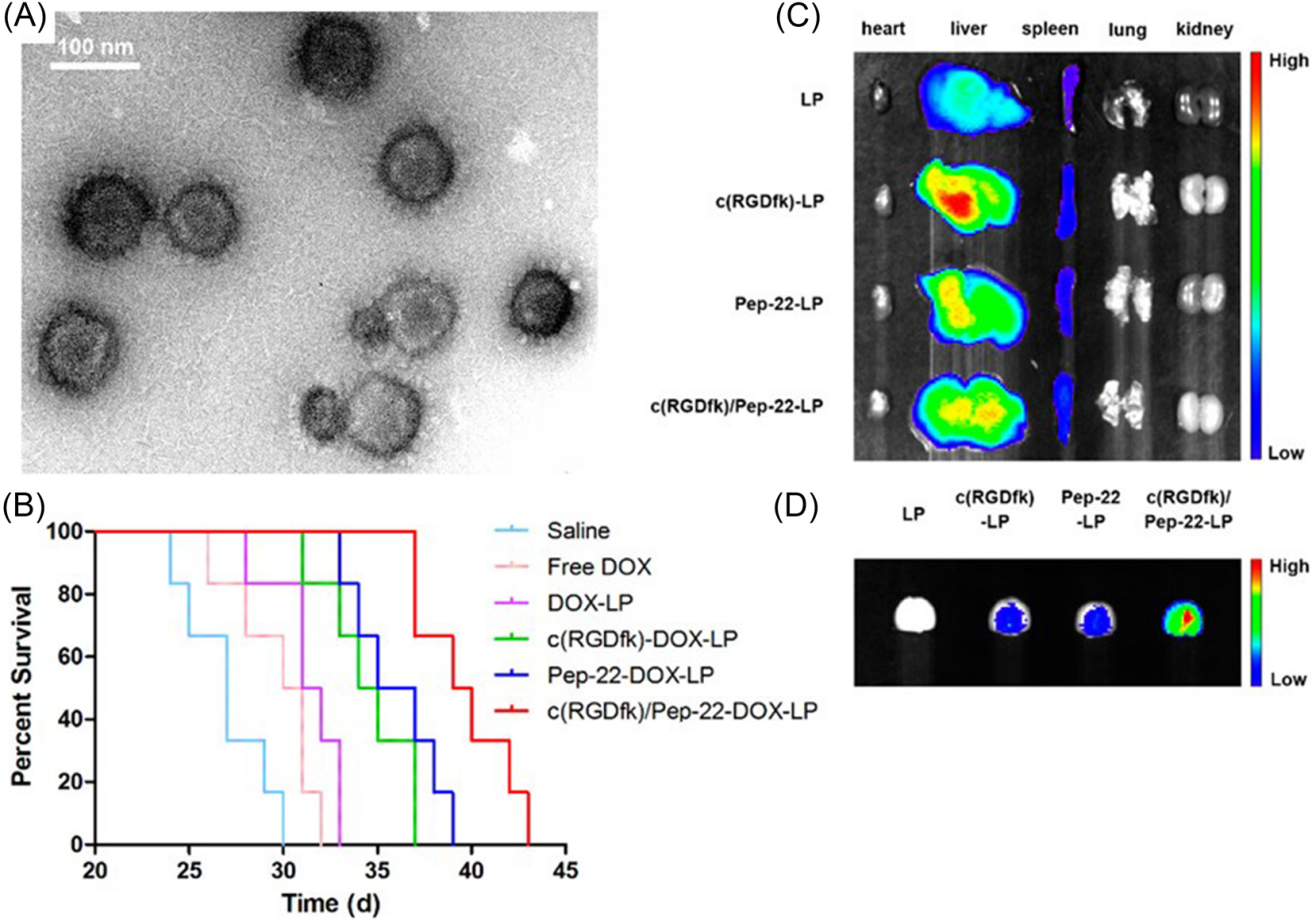
(a) Transmission electron microscopy (TEM) was conducted on c(RGDfK)/Pep-22-DOX-LP, indicating the uniform size and morphology of dually modified liposomes. (b) Kaplan–Meier survival curve of glioma-bearing mice when treated with unmodified liposomes LP, liposomes functionalized with the peptide c(RGDfk)-LP or Pep-22-LP, and co-functionalized liposomes with both c(RGDfk)/Pep-22-LP showing that the combination imparts a lower intensity of localization to the liver as well as a greater intensity of localization to the tumor. *Ex vivo* excised (c) organs and (d) brains in mice with glioma after 24 h post-injection. Adapted with permission from Chen *et al.*, ACS Appl. Mater. Interfaces **9**(7), 5864–5873 (2017). Copyright 2017 American Chemical Society.

Similar to the widespread use of linear RGD peptide ligands, cyclic RGD is often used to functionalize liposomes to improve targeting efficacy and stability. Kang *et al.* functionalized sodium borocaptate-loaded liposomes with c(RGDyC) to improve boron neutron capture therapy.^74^ Notably, when compared to control liposomes, the modified nanoparticles were significantly more toxic, with significantly greater delivery efficiency toward α_v_β_3_ expressing cells. Chen *et al.* demonstrated that co-functionalized doxorubicin-loaded liposomes with peptide-22 and c(RGDfK) improved tumor localization *in vivo* with a reduction in the IC_50_ *in vitro* when treating glioma.^75^ They showed that the surface co-modification was not only stable via transmission electron microscopy (TEM) [Fig. 4(a)], but also improved survival time [Fig. 4(b)], decreased liver accumulation [Fig. 4(c)], and increased tumor localization [Fig. 4(d)] in glioma-bearing mice compared to single modified liposomal controls. In a similar approach, Belhadj *et al.* co-modified liposomes with p-hydroxybenzoic acid (pHA) and c(RGDyK) to target both dopamine receptors and α_v_β_3_ integrins, respectively, on the vasculature of the blood–brain barrier (BBB) and blood–brain tumor barrier (BBTB).^77^ In parallel studies, pHA was linked to c(RGDyK) and conjugated to doxorubicin-loaded liposomes with a PEG linker, c(RGDyK)-pHA-PEG-liposome.^78^ Impressively, the results showed a significant increase in the survival time of mice with glioma when treated with functionalized particles (36.5 days) compared to the surface unmodified control (26.5 days). Amin *et al.* modified the peptide ligand to explore a greater hydrophobicity by adding an *N*-methyl, c(RGDf(*N*-methyl)C) and found it to increase the circulation time and decrease binding to normal cells.^76^

#### 3. Non-RGD ligands

A few groups have also experienced success modifying liposomes with non-RGD-based ligands. Zhang and colleagues discovered a novel linear peptide RWrNK, which exhibited twofold higher binding affinity to α_v_β_3_ than RGD and its mimetic peptides, Cilengitide, and demonstrated a higher sensitivity, specificity, and permeability of the BBB and BBTB.^79^ Wu *et al.* of the Liu group first discovered a novel peptide sequence named P1c, sequence CIRTPKISKPIKFELSG, and its ability to target α_v_β_3_-rich tumor cells.^80^ The group continued to examine the sequence, and Xu *et al.* found that P1c and PEG co-decorated liposomes loaded with doxorubicin were able to reduce tumor angiogenesis and significantly inhibit tumor growth, while simultaneously reducing hepatotoxicity *in vivo*.^81^

### F. Other receptors

Many other receptors have been studied as potential targets for peptide-ligand localization. Although less frequently studied, these receptors have demonstrated promising tumor targeting results and are briefly described below.

#### 1. Gastrin-releasing peptide receptor (GRPR)

Gastrin-releasing peptide receptor (GRPR) is another transmembrane protein, a G protein-coupled receptor, which is highly overexpressed in many types of cancer ranging from pancreatic cancer, glioma, and lung cancer.^104–106^ Although there have been many discoveries of peptides to target GRPR, such as BBN_7–14_,^105^ GB-6,^105^ and AN-215,^106^ functionalizing liposomes for localized drug delivery using this receptor has been relatively underexplored. Akbar and colleagues were able to use a D-enantiomeric GRPR antagonist peptide known as cystabn with the γ-amino acid statine (Sta), sequence FQWAVGH-Sta-L-NH_2_, to treat small-cell lung cancer.^82^ When conjugated to DSPE-PEG2000 lipids and formed into liposomes, the nanoparticles demonstrated increased localization to GRPR over-expressing A549 cells *in vitro*.

#### 2. Interleukin-13 receptor α2 (IL-13Rα2)

**FIG. 5.**
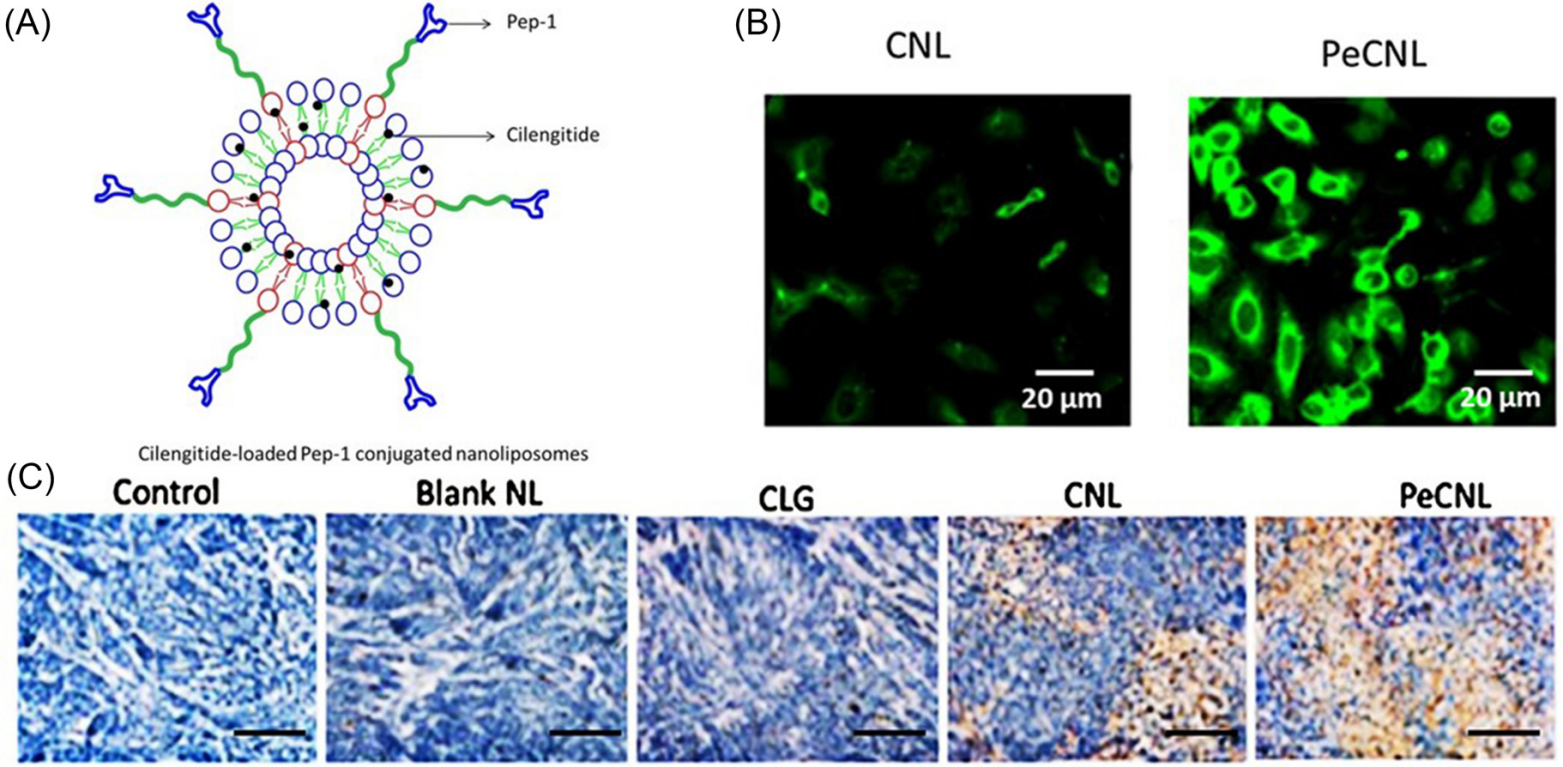
(a) Structural design of Cilengitide-loaded Pep-1-conjugated liposomes. (b) Confocal micrographs of human glioma cells treated with liposomes before (left) and after (right) Pep-1 conjugation. Coumarin-6 (green) was used as a tracking agent. Scale bar = 20 *μ*m. (c) Ki67 immunohistochemical analysis of sectioned tumors from mice. Control-untreated; blank NL-blank liposome; CLG-free cilengitide; CNL-cilengitide loaded liposome; and PeCNL-Pep-1-conjugated cilengitide-loaded liposome. Reprinted with permission from Jiao *et al.*, “Pep-1 peptide-functionalized liposome to enhance the anticancer efficacy of cilengitide in glioma treatment,” Colloids Surf., B **158**, 68–75 (2017). Copyright 2017 Elsevier.

Interleukin-13 receptor α2 (IL-13Rα2) is an overexpressed plasma membrane protein commonly found in glioblastoma multiforme. Using phage display, the peptide sequence CGEMGWVRC, named Pep-1, was discovered to tightly bind to the receptor and penetrate the BBB and BBTB.^107^ Recognizing the applicability of Pep-1 for targeted liposomal delivery, Jiao *et al.* functionalized liposomes loaded with Cilengitide to improve spatial distribution.^83^ Remarkably, cellular uptake was improved from 47.5% to 89.8% when Pep-1 was conjugated to the liposome surface [Fig. 5(a)], and *in vivo* studies exhibited a significant reduction in the tumor volume by targeted formulations in U87-bearing xenograft mice. Notably, Ki67 immunohistochemical analysis of tumor sections from mice treated with Cilengitide loaded Pep-1 liposomes indicated that cell proliferation was greatly reduced compared to controls [Fig. 5(b)]. These advancements in novel peptide design and discovery as seen with Pep-1 are important in targeting IL-13Rα2 for increasing penetrability of the BBB and BBTB to lessen the difficulty of traversing these difficult barriers.

#### 3. Glycoprotein 130 (gp130)

The plasma membrane signal transducing receptor glycoprotein 130 (gp130) is utilized in many cellular functions and has been shown to form a hexameric high-affinity receptor complex with interleukin-11 receptor α with higher expression in glioma cells.^108,109^ Using phage display, Wu *et al.* discovered a 12 amino acid sequence named VTW, sequence VTWTPQAWFQWV, with high binding affinity to the gp130/IL-11Rα complex.^108^ Suga *et al.* used this sequence to functionalize PEGylated liposomes with the previously described (SG)_n_ linker to enhance targeting of glioma cells.^84^ Their results demonstrated a selective association of VTW-K_3_-(SG)_5_/PEGylated liposomes with U251MG glioma cells for enhanced uptake compared to other control ligand sequences.

## III. FUTURE DIRECTIONS

Over the past five years, many advancements have been made regarding chemotherapy-loaded liposomes conjugated with various peptide ligands for tumor-localized therapy. Whether targeting the tumor cell membrane receptors or tumor microenvironment vasculature receptors, success has been demonstrated with increased cellular uptake, tumoral localization, and efficient drug delivery. Although the only clinically approved liposomal therapeutics are either unmodified or PEGylated therapies, numerous clinical trials in all phases are rapidly progressing toward implementing peptide-functionalized liposomes.^25^ Some of the highlights include multi-functional and multi-loaded liposomes for enhanced delivery and combinatorial therapeutic activity, a topic not discussed in detail in this review. These formulations assist in augmenting poor distribution and greatly reduce off-target effects; however, the ubiquitous limitations of systemic clearance by immune cells and multidrug resistance (MDR) remain. Current technologies exhibit difficulties in circumventing limitations of protease degradation, as serum proteins that bind to foreign peptides and proteins bind to and subsequently degrade the sequences conjugated to liposomal surfaces, which decrease drug delivery efficiency.^18^ Furthermore, the discovery of highly specific sequences is limited by large empirical screenings, which are costly and laborious, impeding the profound targeting potential and complete reduction in off-target delivery.

Further investigations in engineering highly efficient, novel, and synergistic therapies are required for the continuous improvement of targeted cancer therapies. For example, structure-vs-function studies should be conducted to elucidate the peptide secondary structure, enabling the design and optimization of sequences to increase binding affinities. Also, expanding the exploration into novel sequences using methods such as microarray chips, phage display, and machine learning will greatly advance the field of peptide targeting. As discussed throughout the review, chirality has shown importance in D-enantiomers increasing serum-stabilizing properties and should be further explored as results contraindicate the expectation for chirality to decrease binding affinity. Synergistic therapies also offer numerous advantages in decreasing drug dose requirements and improving efficacy. In the past, peptides have been shown to synergize with chemotherapies,^46^ which presents a largely underexplored field. Within the next few years, this effort in the field of peptide-modified liposomes will have great success in implementing these technologies in clinical settings to improve cancer patient outcome.

## AUTHORS' CONTRIBUTIONS

M. R. A. conceptualized and wrote the paper; M. R. A., M. J. M., and S. H. M reviewed and edited the paper; and M. R. A. and M. J. M acquired the funding. All the authors have read and agreed to the published version of this manuscript.

## Data Availability

Data sharing is not applicable to this article as no new data were created or analyzed in this study.
